# Chlorine and Nitro-Muriatic Acid

**Published:** 1822-12-01

**Authors:** 


1. *Researches respecting the Medical Powers of Chlorine, particularly in Diseases of the Liver; with an Account of a New Mode of applying this Agent, by which its Influence on the System can be secured.* By WILLIAM WALLACE, M.R.I.A. Member of the Royal College of Surgeons in Ireland; one of the Surgeons to the Charitable Infirmary, Jervis Street; Surgeon to the Dublin Infirmary for Diseases of the Skin; Lecturer on Anatomy and Surgery, &c. Octavo, pp. 164. London, 1822.

2. *Observations from Experience, on the Aid obtained in various Diseases, particularly those incidental to Tropical Climates, by the external Application of the Nitro-Muriatic Acid in a Bath; together with several Cases in which it has been used by the Author with great efficacy; to which is added, the present most approved Mode of mixing the Acids and preparing the Bath.* By PHINEAS COYNE, Member of the Royal College of Surgeons of London; and late of the Honourable East India Company's Service. One vol. 8vo, pp. 140. London, 1822.

WE have reason to think, that the benefits which might have accrued to society at large, from the nitro-muriatic acid, have been prevented, at least for a time, by the indiscriminate application of the remedy to all cases, whether appropriate or not, by the very respectable, but we fear somewhat visionary, author of the proposal. Over Dr. Scott, however, the billows of the ocean now roar unheeded, or his body perchance, is covered by a clod of *foreign* soil; we should not be surprized therefore to see his favourite remedy revive, and come to its proper level among the various means which we employ to mitigate or remove disease.

The great power of external applications over the functions of the internal organs, is now universally admitted, although physiologists may dispute respecting their modus operandi, and the ball of contention may be long bandied about between sympathy and absorption. We believe that both these processes obtain, as the warm bath and mercurial frictions might alone convince any plain, practical, and unbiassed mind. The analogies that exist between the cutaneous and mucous surfaces, whether we consider their structure or their functions, would lead us to a belief in the reciprocal influence which they exert on each other, and which daily observation confirms.

"As instances of these analogies, it may be observed. 1st, They are both endowed with that kind of sensibility, which enables them to bear with impunity the impressions of foreign bodies. 2dly, They are both protected from the influence of these bodies by an inorganic covering: thus, an epidermis covers the cutis, and the chorion of the mucous membranes is covered by an epidermis, by a mucous fluid, or by both. 3dly, They are the seat of all the excretions. 4thly, It is by these surfaces that all substances are introduced into the body from without. 5thly, The capillary portion of their vascular system has the same arrangement. In the foregoing characters they resemble each other, and, by the same characters, they are distinguished from the rest of the organs or tissues of the body; but, perhaps, the most striking proof of their resemblance is to be derived from the circumstance of one being convertible into the other. It is said that some animals of very simple organization can, like the finger of a glove, be turned inside out, and will continue to live; their cutaneous surface becoming mucous, and the mucous assuming the character of the cutaneous. The same transmutation even occurs, to a certain extent, in the human body, when a mucous surface is brought into that situation, which is natural to the cutaneous, or *vice versa.* Thus the mucous membrane of a prolapsed vagina will take on the character of skin; and skin often assumes the nature of a mucous surface, when it is exposed to moisture for a considerable time, as sometimes happens at the folds of the nates, &c. As a further proof of their resemblance, it may be also observed, that the cutaneous surface of the globular hydatid performs the function of both skin and mucous membrane; for, as this animal is not provided with a mouth, all its nutriment must enter by means of the absorbents, which open on its external surface." 5.

When, again, we consider the number, complicacy, and delicacy of the organs over which the mucous membrane is expanded, and the disorders both temporary and permanent, which are frequently produced in their functions, by the application of irritating substances to the said expansions, we should endeavour to limit the exhibition of medicines internally as much as possible, and take all opportunities of seeking for succedanea in external remedies.

Mr. Wallace, after enumerating the various substances which, when applied to the skin, have been found, by experiment, to produce effects (whether by sympathy or absorption) on the internal organs, remarks, that we have every reason to expect a much more decided effect from these external applications, when in a gazeous form, and when the skin is in that state of excitement which necessarily arises from its exposure to a higher range of temperature than under the common modes of application, when the substances used are in grosser forms.

"For, under this exposure, all the vital actions and properties of the organ, both organic and animal, and consequently its sensibility and powers of absorption, are, as far as we can judge, increased. Moreover, it can scarcely be doubted, that medicines, when they are reduced to the state of extreme division, in which state they are, when in the form of gas, will act more efficaciously than when in a grosser state ; and this whether their action result from their direct influence on the skin,* or by gaining admission into the mouths of the cutaneous absorbents. In short, those effects, which hypothesis would lead us to expect from the influence of medicated vapours or gases, applied to the skin, are realized in practice, and perhaps the greatest modern improvement in therapeutics consists, not certainly in the *invention*, but in the perfection and extension of the mode of applying such agents to the surface of the body, for the purpose of influencing the actions of our system." 18.

It is well known that individuals of peculiar idiosyncracy, have skins that resist mercurial frictions, and the same seems to be the case in respect to the nitro-muriatic acid bath. We have used it in a very considerable number of cases, and

---

* "Il est reconnu que ces vapeurs, qui sont pour ainsi dire un être moyen entre l'air et l'eau, sont bien plus pénétrantes et plus actives que ce fluide, lorsqu'il est soumis aux lois de la cohésion ; une grande partie se condense sans doute sur le corps plus frais qu'elles ; mais il n'en est pas de même de toutes ; au moins l'expérience nous apprend que les vapeurs de vinaigre agissent bien plus fortement sur le plomb que le vinaigre sous sa forme fluide." "De la Nature et de l'Usage des Bains, par Henri—Mathias Marcard. Traduit de l'Allemand, par Michel Parant. Paris, 1801," p. 218—19.

have found it apparently inert in some persons, and in others efficient. This seems also to have been the experience of our author, who was thence led to find out the means of employing the remedy, or at least the active principle of it (chlorine) in the form of gas or vapour.

Mr. Wallace accords in opinion with Boerhaave,* and the late Dr. James Curry, that the number of diseases which depend, more or less, on derangements of the biliary system, exceed all others taken conjointly—and he attributes the great success in the practice of Curry, Hamilton, and Abernethy, to their adoption of views more or less in accordance with this principle.

Our author next goes on to the relation of cases in elucidation, arranged according as the liver was primitively or consecutively affected.

Before entering on these cases, it may be proper to state the mode of preparing and applying the chlorine, which our author has used.

For the administration, both partial and general, of diluted chlorine, Mr. Wallace employed, for a long time, Rapou's instrument for *Douches*, and the common fumigating apparatus of this and other countries, which, on the whole answered very well. Latterly, however, he has constructed a very ingenious portable apparatus for the administration of chlorine, and all other vapours and gazeous bodies, a beautiful model of which we have seen in this metropolis. In an hospital, the apparatus can be carried from ward to ward, and yet possesses all the advantages of an immoveable vapour bath.

"The following is the manner in which I procure the gas. I keep always ready prepared a mixture of muriate of soda and of the black oxyde of manganese, well triturated together, in the proportion of three parts of the former and one of the latter; and also a quantity of sulphuric acid, whose specific gravity is to that of water as 1400 is to 1000. By mixing four parts of the compound powder with three parts of the acid, and applying a gentle heat, the gas is quickly brought over, and may be then used, according as it is required for general or partial application. I keep the manganese and muriate of soda ready mixed for greater convenience; and I prefer an acid, diluted as I have mentioned, to that of the strength of either the London or Dublin pharmacopœias, for the purpose of preventing the mixture from boiling over, which often happens, when the acid is used in too concentrated a state." 131.

---

* Boerhaave has said that out of one hundred chronic diseases, there was scarcely one, in which the liver was not affected.

It is impossible to state the quantity of chlorine, which may be necessary for each general fumigation, as that will vary according to the temperature at which it is used, the perfection of the apparatus, the sensibility of the patient's skin, &c. It should always be employed in such quantity as to produce the peculiar sensations which arise from its proper application. If the apparatus be good, no gas will escape to incommode the patient's lungs. If imperfect, it will be very much otherwise. About half an hour will be a medium time for the chlorine fumigation. The general effects of the application of chlorine to the surface were an increased secretion of bile—and that very often without purgation. The proof was, " the extreme bilious character of the fæces." " They are often coloured, as if they were composed almost entirely of the most concentrated biliary matter, and this whether they be solid or fluid." We agree with our author that an increased secretion, and an *accumulation* of bile may take place without necessarily producing purgation; and yet we are convinced that a due secretion and healthy quality of the bile, are the most natural and permanent stimulants for the due action of the intestines.

We shall now proceed to take some notice of the cases detailed by Mr. Wallace.

The first case is that of simple jaundice in a lad aged 19 years, whose skin was completely yellow and stools white for six weeks before he came under our author's treatment. With all the usual symptoms of icterus, he was free from fever, but had " great uneasiness in the epigastrium, (increased by pressure) extending into the right and left hypochondria—dull oppressive, pain in his forehead, &c." After exhibiting colocynth and salts, which only produced some scanty, whitish, sour, and liquid stools, the whole surface of the body, from the neck downwards, was exposed for half an hour to the influence of chlorine, at a temperature of 110°. While in the bath, he said that he felt as if all his skin was pricking by small needles. Next day, as there had been no motion, the purgatives were repeated, and so was the chlorine fumigation. *3d day*. The stools to day were partially tinged with bile. The chlorine repeated. The stools were now spontaneous and very much impregnated with bile. The fumigation was continued, and on the fifth day of application he complained of soreness of his mouth, while the surface of the body presented, in various places, minute bright red papulæ. The icteritious phenomena were now all on the decline. The fumigation was continued for ten or twelve days from the commencement, at which time he was discharged from the infirmary cured.

"It may be easily conceived how much I was gratified by the results of the foregoing case. It was the first, which I submitted to the action of chlorine; and it fully satisfied me, as far as one case could, of the specific influence of this agent on the liver, and of the advantages, which might be expected from its employment." 30.

We have seen a similar effect produced by the nitro-muriatic acid bath. The above case adds one to the great number now on record, where the intimate consent between the cutaneous and hepatic functions is manifested.

The second case is that of an unmarried lady, aged 35, whose liver was evidently much enlarged and indurated, but not very tender on pressure.

"She says she has, at all times, a sensation of dragging in the right side of the abdomen, an incapability of lying on the left side, and a feeling of oppressive weariness in both her shoulders. She is pallid, and appears much emaciated,—bowels constantly demand the influence of purgative medicine to secure an occasional evacuation, which is almost always of a dirty ash-colour,—urine variable, but generally turbid and of a deep brown colour,—skin very harsh, dry, and extremely itchy at night,—feet slightly œdematous,—appetite not very deficient,—digestion much impaired. There does not appear to be much febrile excitement." 32.

This lady had used a variety of remedies, and among the rest mercury, without material benefit.

Our author ordered the general application of chlorine in the dry form, for half an hour each day, at the temperature of 104 Ft. and aqueous vapour and chlorine to be directed in a stream on the region of the liver, for 15 minutes at a time daily. The bowels were also ordered to be cleared daily, by means of pills composed of scammony, aloes, and soap. Having persevered in this plan very steadily for a fortnight, her mouth and throat had become a little sore, the salivary secretion increased, and there was a thick papular eruption brought out over the region of the liver by the topical application of the chlorine. The symptoms were now, however, much ameliorated; and another fortnight's perseverance rendered "the state of this patient wonderfully improved." In short, she completely recovered, though it took a considerable time for that purpose.

Although we have no doubt that the use of the vapour bath proved a powerful auxiliary in the above case, yet knowing as we do, what a long course of purgatives medicines will effect in such cases, we must hesitate in considering the history as so very "illustrative of the great and salutary powers of the chlorine in chronic enlargements of the liver," as our author is disposed to pronounce it. All we can say, however, is—"valeat quantum valere debet."

Our author has since met with several analogous cases in hospital practice, and with similar success.

"What, it may be asked, was the *immediate* cause of the favourable result? I would answer, partly the specific influence of the chlorine on the action of the liver; and partly the local irritation, which it produced in the skin covering this organ. The very seasonable return of the menstrual discharge, and the preternaturally lax state of the bowels, which supervened during the treatment, and which were, no doubt, the consequence of it, had also a very considerable, and a very beneficial influence, in the removal of the disease." 38.

Mr. Wallace believes, and we agree with him, that chlorine exerts a specific influence on the hepatic functions—that is, it increases the biliary secretion.

"We know," says he, "from general experience, that one of the most efficacious ways of subduing a tendency to disorganization, or even an actual alteration in structure, of a glandular part, is by increasing and modifying its secretory actions. In this way, then, do I conceive that the remedy acted, at least in part, in the foregoing case." 38.

Mr. Wallace thinks, that practitioners are not sufficiently aware of the benefit to be derived from "a continued irritation of the skin, or a discharge from this organ, for the relief of deeply seated viscera when affected by chronic disease."

Our author relates an interesting case of a lady, who had suffered, during a space of four years, from a severe periodical pain, apparently seated about the cartilages of the ribs, on the right side, which recurred every two or three days, with harrassing violence, to be mitigated only by opium, the bent-forward position, and pressure. As long as the pain lasted, there was generally nausea, and sometimes vomiting. The liver felt tumid in the epigastrium, but did not descend perceptibly below the ribs—appetite bad—digestion painful, with flatulence and acidity—bowels irregular—stools unnatural—urine scanty and turbid—tongue foul in the mornings. She had been under many respectable practitioners, and the resources of medicine had been tried without success.

On the succeeding day, our author personally witnessed one of these agonizing attacks, and immediately administered a dose of laudanum, and ordered hot fomentations, after which, bitters and aloetic aperients were prescribed daily. He moreover directed the general application of chlorine, in conjunction with watery vapour, at the temperature of 98°, for twenty minutes daily, and a similar topical application to the region of the liver, for ten minutes at a time, every second day. This plan was continued, with very little modification, for six weeks, when her mouth and throat became sore, with an increased discharge of saliva, considerable cutaneous irritation over the region of the liver, and, also, a general papular eruption over the body.

"The amendment has been such as must appear extraordinary to every one acquainted with the obstinacy and intractable nature of such cases. From five days after she had begun the employment of the chlorine, there was a sensible diminution in her sufferings; and, for the last eight days, she has not had any return of the periodical pain. The pain seated in the epigastrium is almost entirely removed. There is also, in other respects, a very material improvement. Her colour is much less yellow, and she appears to be regaining her flesh." 43.

It is evident that the means employed here, independently of the chlorine impregnation, were well calculated to mitigate the symptoms, if not remove the disease. We have seen several cases of this kind, which we at first attributed to gall-stones, but which we afterwards thought were owing to some spasmodic affection about the gall-ducts and duodenum. They were relieved, and most of them cured, by a liberal exhibition of opiates and aromatics at first, with the warm bath, and, subsequently, alterative aperients, together with the nitro-muriatic acid bath. There is a lady of our acquaintance, who always keeps the ingredients for preparing the nitro-muriatic bath in the house, and whenever she has any warning of these attacks, she uses the bath to the feet and legs every night for a week or so, and thus wards off the disease. The perceptible effects of the bath, at these times, are an increase of yellowness in the stools—in fact, an increase of the biliary secretion, and a relaxed state of the bowels.

After relating another case in which the liver was obviously affected, and in which the chlorine was successful, our author goes on to the subject of what we might term "masked hepatic affections," or cases in which the principal expression of the disease is at a distance from the true seat of it. Those most acquainted with hepatic diseases, whether structural or functional, well know how anomalous are often the symptoms, and how liable men are to be led astray, who expect to always find the nosological characters assigned to diseases by systematic writers, as a sure guide in their practice.

"The sympathetic affections, which result from hepatic disease, are so frequent, that I believe I may, with great truth, affirm, that there is scarcely an important organ in the body, whose functions are not occasionally disordered by a wrong action of the liver. Is there any practitioner, who cannot immediately recall to his memory numerous instances of disorder of the brain, of the lungs, of the heart, stomach, bowels, uterus, kidneys, skin, &c. &c., which have sprung from this source?" 53.

The sympathetic connexion between the liver and heart is well known. They affect each other mechanically, as well as sympathetically, more especially when in a state of disorder. We shall select a case or two from our author by way of illustration.

*June* 3, 1821. Mr. G. about 35 years of age, of active disposition, but sedentary occupation, full, sanguineous habit, but lately of sallow complexion and much emaciated.

"He complains of distressing and almost constant palpitation, with an occasional tendency to syncope. The former is greatly increased by bodily exertion or mental anxiety, but the latter frequenly comes on without any apparent cause. His pulse, at present, is irregularly intermittent, and not more than seventy-four in a minute. He also complains very much of a sensation of constriction round the middle and lower part of the thorax, accompanied by a numbness, which extends from his shoulders down his arms to his elbows. This comes on only at intervals. He cannot bear the most trifling fatigue; not only on account of its producing violent palpitation, and most distressing sinking sensations in the region of the heart, but also on account of the profuse and exhausting perspirations which attend it." 58.

These complaints were of about a year's standing, but his health has not been good for three or four years. He had an attack of acute hepatitis three years since, for which he had been bled copiously. He cannot lie on the left side at night, nor bear his clothes of a moderate degree of tightness round the hypochondria. The epigastrium appears full, and tender to the touch—bowels very irregular, but generally constipated, at which times the cardiac symptoms are most troublesome. The urinary discharge is generally turbid, skin unperspirable, and often feeling like a piece of cold marble.

Our author ordered a purgative of salts and senna, which brought away copious jellatinous stools, but no fœcal matter. He was then directed to take pills composed of aloes, scammony, rhubarb, and essential oil of cinnamon, every second night, while the chlorine, in conjunction with aqueous vapour, was applied to the general surface for half an hour daily, at a temperature of 110°. He remained under this treatment for nearly six weeks, during which there was a gradual improvement in the appearance of his discharges, they becoming, at last, perfectly natural and regular, without the assistance of medicine; "and exactly in the same proportion, the symptoms which depended on derangement of the heart also subsided." The chlorine rash (hereafter to be described) was pretty severe. The gentleman has continued in perfect health afterwards.

We agree with our author that the above was a case of functional disorder of the heart, brought on, and kept up by, a disordered action of the liver and other digestive organs. But as the eccoprotic medicines combined with the chlorine, were very suitable in themselves, we cannot be safe in attributing the whole of the cure to the latter medicine. At the same time, we have no doubt that the chlorine was mainly instrumental in the patient's recovery.

Our author, since his opportunities of observing cutaneous diseases have become extensive, has had his attention forcibly drawn "to their connexion with the state of the liver." "In fact, (says he) I have so frequently remarked this connexion, that I now never think of entering on the treatment of any disease of the skin, until I have particularly enquired into the state of the liver." Mr. Wallace relates an interesting case of ecthyma in illustration of this connexion. The ecthymatous eruption is one which depends almost always on constitutional disorder, and is frequently mistaken for secondary syphilitic eruption, more especially as mercury has generally a beneficial influence on the disease. "But this, I conceive, (says Mr. W.) is attributable to the action of the mineral on the liver, whose disorder is the source of the cutaneous affection." Our author makes many sensible and acute observations on those affections of the liver which are determined by lesions of other, and sometimes distant organs—especially injuries of the head. To these observations, we would recommend our reader's attention; but we shall quote one case in illustration.

"A man, who had been for many years subject to a violent oppressive pain in the upper part of his forehead, so circumscribed, that it could be covered by the end of a finger, received a wound exactly in the situation of the part which was the seat of pain. The wound was not more than three-fourths of an inch in length. The periosteum was divided, but the bone was not injured. The wound was a simple incision, unattended by any contusion, and inflicted in such a manner that there could not have been the slightest concussion. Immediately after the receipt of the wound, the pain, which had for years almost incessantly harrassed him, suddenly disappeared. He therefore considered the occurrence of the wound a most fortunate event; and, at his desire, it was dressed in such a way as to prevent its lips being brought together. In the course of two days, the system began to sympathize very remarkably. A sensation of horripilation and great sensibility of the surface of every part of the body set in. In twenty-four hours after this, the scalp and face were attacked by an œdematous swelling; but the skin was not, at first, in any way red. On the contrary, it was rather pallid; but the scalp became so extremely sensible, that not the least pressure could be borne. About this time, that is, three days after the wound of the head, the entire surface appeared perfectly jaundiced; indeed as yellow as I have ever seen it in any case of pure icterus. Matter formed in various parts over the scalp, and in the eyelids. The discharge from his bowels became whitish, not having the slightest appearance of bile; but his urine assumed so much the aspect of this fluid, that it seemed more like the contents of the gall bladder than any thing else. It was, nevertheless, sufficiently copious. A low muttering delirium now commenced, which soon became violent, and was particularly marked by an unrestrainable propensity to tear the hair from his head, to pull with his nails at his lips, to beat his sides with his arms, and to gnash with his teeth. Whenever he sunk into a slumber, there was an almost continued grinding of his jaws, resembling that which so often occurs in children, from the disordered state of the alimentary canal and its appendages. He died (very much like a person in typhus) on the tenth day after the receipt of the wound, and seven days from the commencement of the symptoms of jaundice. On dissection, the liver and kidneys were the only parts observed to be morbidly affected. The structure of the former was perfectly natural; but there had been a complete suppression of the secretion of bile. The gall bladder was full of a mucous fluid, much resembling the white of an egg, transparent, colourless, and entirely void of any bitter taste. The kidneys were greatly enlarged. This enlargement appeared, however, to be the result of recent sanguineous congestion. Their pelvis contained the same kind of fluid which had been evacuated as urine. It was, however, in a more concentrated state, and of an extremely bitter taste. The contents of the cranium were in no way diseased. Whether the cessation of the secretory powers of the liver was, in the preceding case, the immediate cause of death, I shall not take upon me to determine. It must, however, be considered as a very remarkable example of the power of external injury to cause very serious functional disorder of the biliary organ." 83.

We must pass over the remaining cases detailed in this volume, in order to convey to our readers some of Mr. Wallace's observations on the "mode of action" of chlorine on the system, when in a state of health. For the purpose of ascertaining this mode of action, our author submitted himself and some of his pupils to the influence of the remedy; and from these trials principally, the following facts and conclusions have been noted and drawn.

1. *Skin.* If the cutaneous tissue be exposed, in a proper apparatus, to the action of chlorine sufficiently diluted with air or aqueous vapour, of the temperature 110° sensations will be excited, in ten or twelve minutes, on different parts of the surface, resembling those produced by the stings or bites of very minute insects, which sensations gradually increase in number but not in severity, and, at length, create a desire to slap with the palm of the hand, the parts which are stung.

These stinging sensations never continue troublesome after the person has come out of the bath; but they are generally succeeded by an increased degree of itchiness and slight smarting which, however, terminate before the patient is dressed. Our author has reason to think that, the cutaneous structure remains more sensible to impressions, for a considerable time after each operation. An increase of perspiration is another immediate effect of the chlorine application, commencing, in general, with the stinging sensation, and in most instances, proving very copious. This perspiration, Mr. Wallace asserts, "is always more copious than would be produced by the same degree of heat, either alone or combined with aqueous vapour." Our author found himself more disposed to perspire on the night succeeding the chlorine bath, than usual; and to this property of the remedy, he attributes much of the advantages derived from its use.

The most remarkable effect, perhaps, of the bath, is the chlorine rash—an eruption of the most minute papulæ, appearing on all parts of the body, but more particularly on the back, loins, breast, abdomen, and arms. The occurrence of this eruption is always to be considered a beneficial event. Mr. W. has very rarely observed the papulæ to pass into suppuration or vesication. In local applications of the chlorine gas, the skin assumes a red appearance, and, if the application be continued, considerable pain is the consequence. The pain and redness increase, the skin becomes elevated and tumid, resembling, very much, the aspect which the integuments of the face assume under erysipelas, succeeded by a sense of soreness and as if the parts had been contused. These sensations continue for several days, the skin still appearing to be deeply affected, and, at last, being replaced by itchiness and desquamation of the cuticle.

"From what has been said it appears, that the immediate effects which arise from the application of diluted chlorine to the skin are an exaltation of its sensibility and the excitation of peculiar sensations, an increase of its secretions, a preternatural accumulation of blood in its capillaries, and finally an elevation of its natural temperature; from all of which we are authorized to conclude, that its functions and vital properties are brought into a state of excessive preternatural excitement, which continues for a *considerable time after each operation.*" 108.

II. *Mucous Membranes.* The action of chlorine, our author believes, on the mucous membranes, is somewhat analogous to that which it exercises on the skin. "Thus, when a person is under the influence of this agent, there is an alteration in the quantity and quality of all the secretions which are performed on these membranes, but more particularly of those of the biliary and salivary glands, and of the urinary and genital organs, &c."

III. *Respiration and Circulation.* These are increased by exposure to the influence of the chlorine, but whether owing entirely or not to the heat, our author is not able to determine. Its action on the brain and nervous system does not appear to have been clearly ascertained by our author. He is, however, far from limiting the action of chlorine to these its *sensible* effects.

"I am, indeed," says he, "certain that much of its value in chronic disease is owing to an action, which, although most gradual in its operation finally accomplishes a general and complete change in the organization." 111.

Our author makes many important observations on the utility of this medicine in hepatic diseases in particular; but we shall only allow ourselves to make one short extract more.

"To communicate, in a single sentence, a rule for the regulation of our conduct respecting the employment of chlorine in diseases of the liver, I may remark, that, *in all cases of hepatic disease, which consist in a torpid or wrong action of the secretory powers of the biliary organ, but which are not attended by active inflammation, it will be found an invaluable remedy, and may be boldly used with well grounded expectations of success.* It is scarcely necessary to make any remarks on the frequency of such states of the liver, and on the numerous anomalous symptoms to which they give rise." 119.

We think we have extracted enough from Mr. Wallace's work, to induce the medical officers of public institutions (who have the best means) to give trial to the chlorine vapour bath, in the diseases pointed out by our author. And if it shall answer the expectations here excited, Mr. Wallace will have deserved well of the profession. Till this trial be made, we shall abstain from all further comment on the work before us.

Our limits are so far overstepped, that we can dedicate but a few lines to the second work on our list. Mr. Coyne has long used the nitro-muriatic acid bath, both in India and in this country; he is, therefore, much attached to the remedy. He enters into a defence of the nitro-muriatic acid bath, against those who opposed or condemned the measure, (most of them indeed, without giving it any, or, at least, a sufficient, trial,) and has published several cases, illustrating the beneficial effects resulting from its application. As the Treatise is "not intended solely for the perusal of gentlemen of the medical profession," we shall abstain from any criticism, especially as we are unable to enter, at the same time, into an analysis of the work.

